# The VA National Teleneurology Program (NTNP): Implementing Teleneurology to Improve Equitable Access to Outpatient Neurology Care

**DOI:** 10.1007/s11606-023-08121-7

**Published:** 2023-06-20

**Authors:** Jayne Wilkinson, Laura Myers, Joanne Daggy, Holly Martin, Grace Bastin, Ziyi Yang, Teresa Damush, Aditi Narechania, Steve Schriber, Linda S. Williams

**Affiliations:** 1 Corporal Michael J Crescenz VAMC, Philadelphia, USA; 2 Department of Neurology, University of Pennsylvania, Philadelphia, USA; 3 Richard L. Roudebush VAMC HSR&D EXTEND QUERI, Indianapolis, USA; 4 Department of Biostatistics, Indiana University School of Medicine, Indianapolis, USA; 5 Regenstrief Institute, Inc., USA, Indianapolis, USA; 6 Department of Medicine, Indiana University School of Medicine, Indianapolis, USA; 7 Jesse Brown VAMC, Chicago, USA; 8 University of Illinois Chicago, Chicago, USA; 9 Northwestern University, Evanston, USA; 10 Tibor Rubin VAMC, Long Beach, USA; 11 University of California, Irvine, USA; 12 Department of Neurology, Indiana University School of Medicine, Indianapolis, USA

## Abstract

**Background:**

Telehealth is increasingly utilized in many healthcare systems to improve access to specialty care and better allocate limited resources, especially for rurally residing persons who face unique barriers to care.

**Objectives:**

The VHA sought to address critical gaps in access to neurology care by developing and implementing the first outpatient National Teleneurology Program (NTNP).

**Design:**

Pre-post evaluation of intervention and control sites.

**Participants:**

NTNP sites and VA control sites; Veterans completing an NTNP consult and their referring providers.

**Intervention:**

Implementation of the NTNP at participating sites.

**Main Measures:**

NTNP and community care neurology (CCN) volume of consults before and after implementation; time to schedule and complete consults; Veteran satisfaction.

**Key Results:**

In FY2021, the NTNP was implemented at 12 VA sites; 1521 consults were placed and 1084 (71.3%) were completed. NTNP consults were scheduled (10.1 vs 29.0 days, *p* < 0.001) and completed (44.0 vs 96.9 days, *p* < 0.001) significantly faster than CCN consults. Post-implementation, monthly CCN consult volume was unchanged at NTNP sites compared to pre-implementation (mean change of 4.6 consults per month, [95% CI − 4.3, 13.6]), but control sites had a significant increase (mean change of 24.4 [5.2, 43.7]). The estimated difference in mean change in CCN consults between NTNP and control sites persisted after adjusting for local neurology availability (*p* < 0.001). Veterans (*N* = 259) were highly satisfied with NTNP care (mean (SD) overall satisfaction score 6.3 (1.2) on a 7-point Likert scale).

**Conclusions:**

Implementation of NTNP resulted in more timely neurologic care than care in the community. The observed significant increase in monthly CCN consults at non-participating sites during the post-implementation period was not seen at NTNP sites. Veterans were highly satisfied with Teleneurology care.

**Supplementary Information:**

The online version contains supplementary material available at 10.1007/s11606-023-08121-7.

## INTRODUCTION

Accessing neurological care presents numerous challenges for patients often facing chronic, disabling conditions. The nation-wide scarcity of general neurology providers^1^, coupled with physical, geographical, and financial barriers to care, creates an enormous hurdle to obtaining timely neurological assessment and introduces a variety of disparities in access to care. Studies show that access to neurological care improves clinical outcomes^2,3^ and therefore increasing and facilitating access is imperative, particularly in rural, underserved areas.

Programs and processes that alleviate these barriers are a priority for healthcare systems, one emphasized by the SAR-CoV-2 pandemic which greatly impaired access to traditional face-to-face healthcare. While telehealth modalities have been utilized in neurological care for many years, larger-scale, sustainable programs in the pre-COVID era were uncommon.^4,5^ In fact, many healthcare systems did not institute telehealth programs until the global pandemic. The Veterans Healthcare Administration (VHA), however, has historically led the way in supporting telehealth programs (beginning in 1959), and conducting more than 2.5 million telehealth visits in 2019; this effort expanded during the pandemic with an increase of nearly 800% in telehealth visits by May 2020.^6^

In 2019, VHA implemented the *Congressional Mission Act of 2018* to increase Veteran access to healthcare by offering community care for eligible Veterans.^7^ Eligibility for specialty care was outlined as a drive time  >60 min, or a wait time  >28 days from the date of appointment request to see a VHA provider. Despite this additional resource, the general shortage of neurologists, even in the community, dampened its impact for neurology care as community wait times far exceeded the desired range of within 28 days.

In January 2020, VHA funded the National Tele-Neurology Program (NTNP), a national-scale telehealth program that leverages technology to bring neurological services to some of the most rural areas of the country. NTNP is a patient-centered, innovative approach to expanding timely access to ambulatory neurological care for rural Veterans that has built a virtual cohort of physicians, nurses, and telehealth staff in a flexible, cost-effective hub-spoke model that uses synchronous (video) and asynchronous technologies. The program recruited and coordinated providers from across the country to work remotely and, in turn, successfully implement services at multiple spoke sites. NTNP provides outpatient general neurology care for all neurological conditions and, although it is not scaled to provide total neurological care needed at every participating site, could be expected to impact the volume of community care neurology (CCN) consults placed at participating sites. We undertook this analysis to investigate the impact that implementing NTNP had on both the timeliness of access to neurological care, as well as the volume of community care neurology consultation among participating and non-participating VHA sites.

## METHODS

### Design

Guided by the RE-AIM framework^8^, an implementation framework that organizes the evaluation of programs along the domains of Reach, Effectiveness, Adoption, Implementation, and Maintenance, we conducted a prospective implementation evaluation of the VA NTNP, including three key analyses related to the impact of NTNP on Veteran access to outpatient neurological care: (1) assessment of consultation volume and Veteran and referring provider satisfaction and experience; (2) a comparison of consult scheduling and completion times between NTNP and community care neurology at each site; and (3) modeling the volume of Community Care Neurology consultations at NTNP and non-NTNP sites in the post-implementation versus pre-implementation time period.

### Setting

The VA NTNP was funded by the Office of Rural Health (ORH) with start-up activities in FY2020 and the first clinical implementation in October 2020. During FY2021, the NTNP was implemented at 12 VAMCs beginning with seven neurologists providing 3.75 FTEE; sites joining after FY2021 are not included in this analysis.

Synchronous telehealth visits are conducted using either video to home (VA Video Connect, VVC) or video in an outpatient clinic (Clinical Video Telehealth, CVT). Veterans can choose between NTNP and other neurology services for which they may be eligible, including CCN. All data analyzed in this project were collected for operational and quality improvement purposes as part of the NTNP ORH evaluation; this project was approved as operational (non-research) by the VA and the Indiana University IRB (see signed memo of understanding, Appendix A).

### Subjects

Subjects in this analysis include Veterans with an NTNP initial consultation in FY2021. Sites for NTNP were selected for implementation by NTNP operational leadership in FY2020 based on access to Neurology care, including wait times, and local neurology FTE data. We identified seven control sites for this analysis based on similar VA neurology FTE to NTNP sites (no or  <1.0 VA Neurology FTE in FY2021) and FY2020 or FY2021 contact with NTNP leadership demonstrating interest in possible future participation in the program but with no implementation of NTNP as of the end of FY2021.

### Consultation and Administrative Data

Data on NTNP and Community Care neurology consultations were obtained from the VA Corporate Data Warehouse, including consult status and dates of consult activity; consults were labeled discontinued if they had either discontinued or canceled final status. Consultations for neurological procedures or diagnostic testing were excluded. Total NTNP encounter data (initial patient and follow-up completed visits) and local Neurology availability (full time equivalent (FTE) for FY2020 and FY2021 at each site) were obtained from VA operational and workforce reports. We calculated days from the date the consult was placed to the date it was scheduled and from the date it was placed to the date it was completed. The neurologists’ primary diagnosis for the NTNP visit was assessed by primary ICD10 code assigned to the encounter; these were grouped into common neurological disease categories based on clinical review (e.g., headache, movement disorders, dementia); ICD10 codes indicating a symptom and not a specific diagnosis (e.g., “dizziness”) were grouped into a “Symptom” category. Rurality of each patient was classified using VHA designation as Urban or Rural/Highly Rural.

### Veteran and Referring Provider Satisfaction

Veterans who completed an NTNP consult in the first 6 months of NTNP activity at their site were eligible for a patient satisfaction interview. We attempted three calls with each Veteran seen in the first 3 months of program implementation and a random 50% of those seen in months 4 through 6 at each site. Questions about satisfaction, similarity of the visit to an in-person visit, and likelihood of recommending a teleneurology visit were asked. These questions were individually answered using a 7-point Likert scale where a higher score indicated greater satisfaction. Provider satisfaction was assessed with an emailed survey sent via VA REDCap within 1 week of completion of the NTNP consult; if the provider had answered an NTNP survey in the preceding 30 days the current consult was excluded. Provider surveys were also completed for the first 6 months after program implementation at each site. Providers were asked about overall satisfaction, whether the consult answered their question, and clarity of the consult; items were rated on a 1–10 scale, higher indicating greater satisfaction.

### Descriptive and Statistical Analysis

#### Program and Participant Characteristics and Satisfaction

We described the characteristics and counts of each Veteran with an NTNP consult and the characteristics of each NTNP consult placed including final status and diagnosis for each completed consult. We calculated the mean and standard deviation for three key satisfaction questions for Veterans with a completed NTNP consult and providers who placed a completed NTNP consult.

#### Access Analyses

The time in days to schedule a consult (date scheduled minus date placed) and complete a consult (date completed minus date placed) between NTNP and CCN consults were compared using a Wilcoxon rank-sum test, excluding patients that had both an NTNP and a CCN consult.

To determine the impact of the NTNP program on the number of CCN consults, we conducted a site-level analysis examining the difference in monthly volume of CCN consults before and after NTNP implementation between NTNP and control sites. We included 11 of the 12 NTNP sites; one site only had 1 month of post-implementation data in FY2021 and thus was excluded.

Two fiscal years of data (24 months) were collected for each of the 18 sites; FY20 and the first month of FY21 served as the pre-implementation period for both NTNP and control sites. We considered defining the pre-implementation period based on the date each NTNP site went live but since CCN contracts and policies typically change with VA the fiscal year, having different FYs in the pre-NTNP period might introduce unwanted variation in the baseline CCN volume estimation between sites. We defined the NTNP post-implementation period by site from the month each site began NTNP (starting in month 14) through month 24; for all control sites months 14–24 served as the post-implementation period (see Appendix B, Supplementary Fig. 1). As a preliminary analysis, we used a Wilcoxon signed-rank test to examine the within-site changes in CCN consults in NTNP sites and control sites. Separate within-site sensitivity analysis was done including the previously excluded 12th site which did not change the results. A generalized linear mixed model was then fit to the number of monthly CCN consults for each site. Since the number of CCN consults represents a count, we evaluated both a Poisson and negative binomial distribution with log link. Effects included in the model were as follows: an indicator for the month the program went live (for NTNP sites) or the month the first NTNP site went live (for control sites), a site indicator for NTNP versus control, month (as continuous), and all the 2- and 3-way interaction terms. The model also included local Neurology FTE available at each site in each fiscal year as a covariate and random site-level intercept and slope terms. Estimation was conducted using maximum likelihood with Laplace approximation and appropriate model-fit indices were evaluated. From the fixed effects of the model, we aimed to determine if there is a difference between NTNP and control sites in the level change (i.e. shift up or down) at the time the NTNP program started. We also obtained the main contrast of the difference in mean number of CCN before and after the start of the NTNP program in NTNP sites relative to control sites.

Supplementary analyses included obtaining individual contrasts from the model comparing each NTNP site to control sites during the corresponding time period in which that NTNP site was active. As there are 11 contrasts, significance was defined as a *p*-value < 0.0045 to maintain familywise error at 0.05. For each site, a plot was created with the mean observed and model-predicted number of CCN consults for each month (Supplementary Fig. 2).

## RESULTS

### Descriptive Data

**Figure 1:**
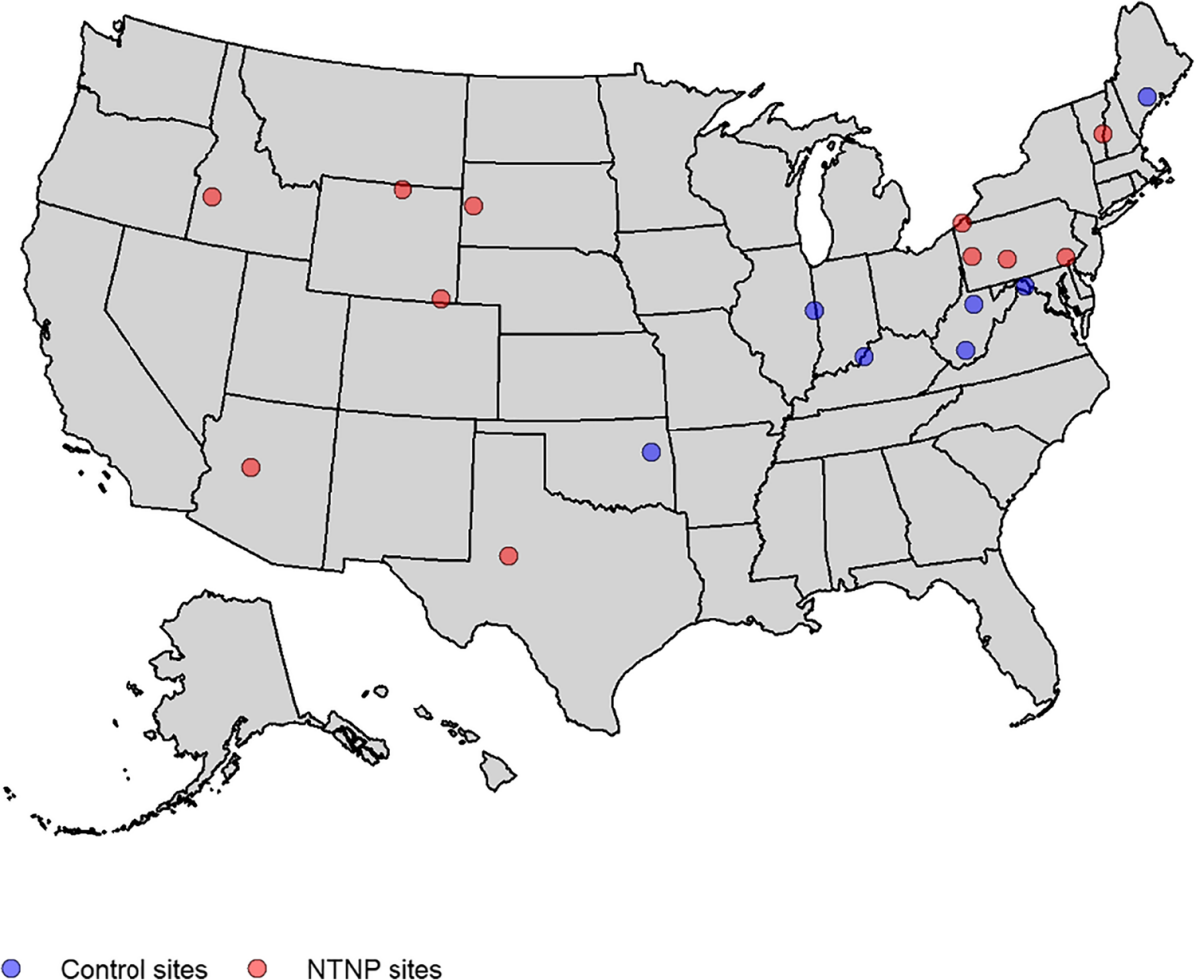
Map of NTNP and control sites.

In FY2021, NTNP was successfully implemented in 12 VAMCs (Fig. 1); six sites had limited local VA outpatient neurology care and six had none. The time from initial site meeting to program launch averaged 92.3 days, with a range from 21 to 197 days. The total volume of new patient consults placed was 1521: 71% were completed, 28% discontinued (either before the scheduled date or after a no show), and 0.2% remained pending/scheduled for completion in FY22 (Table 1). Rurally residing Veterans made up 51.5% of all consults placed. The most common diagnosis categories after the first NTNP visit were headache, symptoms, and movement disorders.

**Table 1:** FY21 NTNP Site, Consultation, and Access Data

Site implementation data	
Number of sites	12
Time to implementation, *mean (SD) days*	92.3 (49.7)
New patient consult data	
Total placed	1521
Completed	1084 (71.3%)
Discontinued	431 (28.3%)
Pending/scheduled	4 (0.26%)
Total encounters (new and follow-up visits)	1306
Neurology diagnosis	
Headache	273 (25.2%)
Symptoms*	162 (14.9%)
Movement disorder	158 (14.6%)
Neuropathy/radiculopathy	99 (9.1%)
Dementia	93 (8.6%)
All others	299 (27.6%)
Consultation access	
Time to schedule, *mean (SD) days*	
VA NTNP	10.1 (15.3)
Community care	29.0 (30.1)
	*p* < 0.001**
Time to complete, *mean (SD) days*	
VA NTNP	44.0 (29.4)
Community care	96.9 (67.7)
	*p* < 0.001**
CCN monthly consult volume change post-implementation, *Mean [95% CI] consults*	
VA NTNP sites (*N* = 11)	4.6 consults [− 4.3,13.6], *p* = 0.413***
Control sites (*N* = 7)	24.4 consults [5.2, 43.7], *p* = 0.016***

^*^ “Symptoms” as a category refers to an ICD-10 code that reflects a symptom (e.g., dizziness) and not a diagnosis. “All others” refers to the sum of all other neurology diagnosis categories

^**^Wilcoxon rank-sum test

^***^Within-site difference in monthly CCN consults post-NTNP implementation–pre-NTNP, Wilcoxon signed-rank test

### Satisfaction Data

Of the 540 Veterans eligible to be called after their consult, 264 (48.9%) completed a phone interview and 259 gave complete responses. Mean satisfaction scores (range 1–7) were high across all sites; combining sites, the total program overall satisfaction mean score was 6.3 (SD 1.2), likelihood of recommending NTNP score was 6.3 (SD 1.3), and the extent to which the teleneurology visit was like an in person visit mean score was 5.6 (SD 1.4). Referring providers were similarly highly satisfied: of the 146 providers that returned a survey after their consultation was completed (25% response rate), overall satisfaction (1–10 scale, mean 8.9, SD 1.7), the extent to which the consult addressed their question (9.0, 1.7), and the clarity of the neurologic plan (9.0, 1.6) were all rated highly.

### Access Data

The average time from NTNP consult placed to scheduled was 10.1 days and from placed to completed was 44 days, which was significantly faster than care in the community (Table 1).

**Figure 2:**
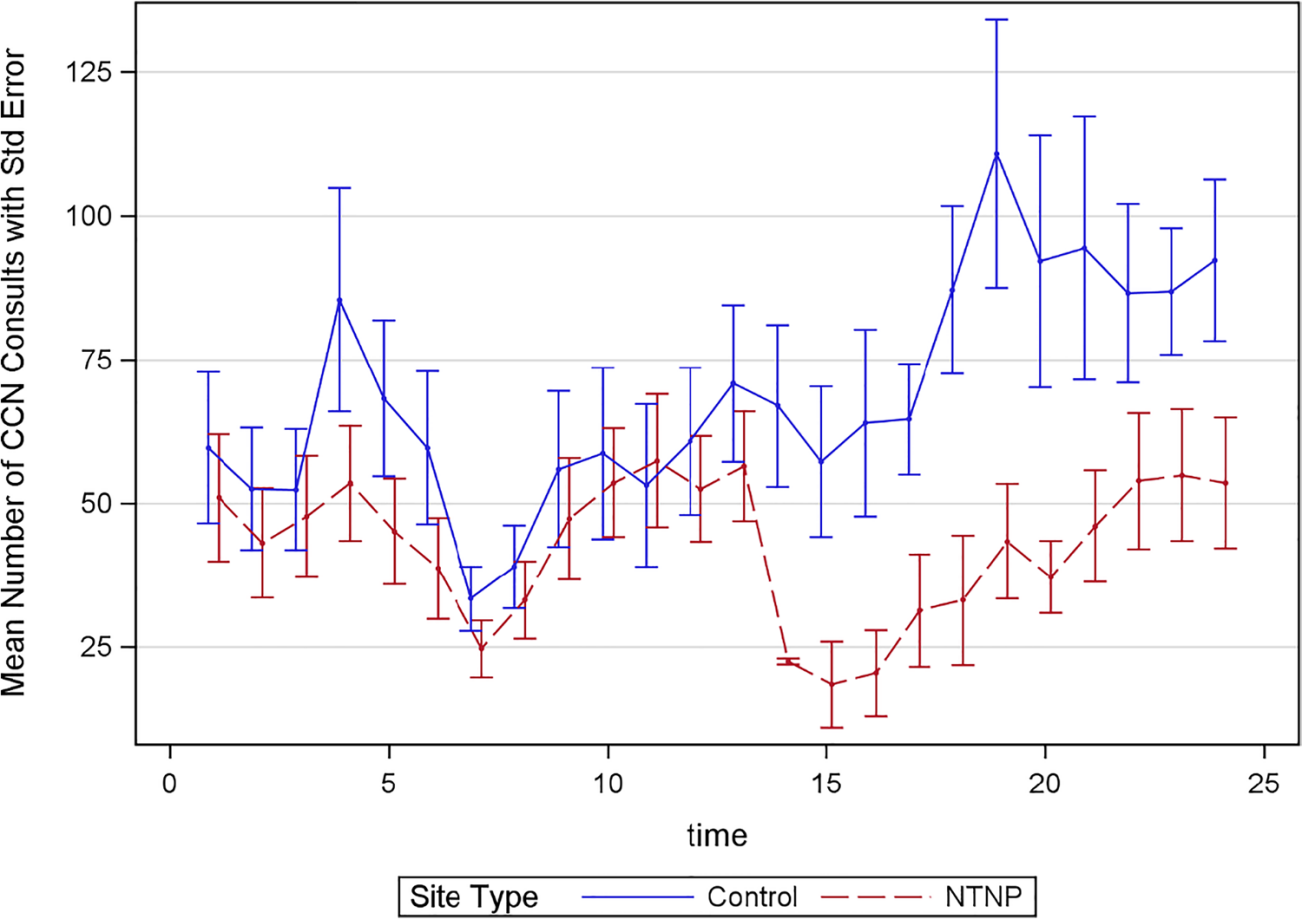
CCN monthly consult volume pre- and post-NTNP implementation. NTNP sites are shown in the red dashed line, control sites in the blue solid line. Months 1–13 are the baseline period (October 2019 through October 2020). Month 14 is the first in which the first NTNP site was active for the entire month.

Figure 2 shows the mean and standard error of monthly CCN consults by month for the 11 NTNP sites and 7 control sites. Additional site-level descriptive statistics for CCN for the pre- and post-NTNP time periods are also available (Supplementary Table 1). The first NTNP implementation began in month 14, which also identifies the start of the post-time period for all control sites. The mean difference (post-NTNP-pre-NTNP) in monthly CCN consult volume at the NTNP sites was not statistically significant (4.6 consults [95% CI =  − 4.3 to 13.6], Wilcoxon signed-rank test *p* = 0.413), but the control sites had a statistically significant increase of 24.4 [95% CI = 5.2 to 43.7] CCN consults per month (Wilcoxon signed-rank test *p* = 0.016) in the post-implementation period (Table 1). Both a Poisson and negative binomial distribution with log-link were evaluated but the model fit indicated overdispersion was present based on the Poisson model and the negative binomial had a lower AIC indicated better fit (Supplementary Table 2), thus only the negative binomial model is reported.

Parameters estimated for the negative binomial model fit to the monthly number of CCN are presented in Table 2 (model-fit indices and covariance parameter estimates available in Supplementary Tables 2 and 3). Based on the model, there was not a significant slope change in monthly CCN consults before and after the start of the program (time × live *p* = 0.154) and the slope change did not vary between NTNP and control sites (time × program × live *p* = 0.405). However, the mean change in the level of CCN consults at the time the program went live did significantly differ between NTNP and control sites (program × live *p* = 0.027): contrasts obtained from the model (Supplementary Table 4) indicate control sites went from a mean of 58.6 CCN consults per month in the pre-period to a mean of 85.9 during the post-NTNP period which was statistically significant (*p* < 0.0001), whereas NTNP sites went from a mean of 43.8 CCN consults during the pre-time period to 42.1 CCN consults during the post-time period, which was not significant (*p* = 0.603). Thus, the estimated difference in mean change in monthly CCN consults at NTNP sites was significantly different from the mean change in control sites (*p* = 0.0001) after adjusting for local neurology availability (FTE).

**Table 2:** Fixed Effects from Negative Binomial Model of Monthly CCN Consults

Effect	Est	SE	*t* value	*p*-value|
Intercept	4.0412	0.2178	18.55	<.0001
Local neurology (FTE)	−0.2523	0.2040	−1.24	0.217
Program (NTNP vs control)	−0.3554	0.2572	−1.38	0.168
LIVE	−0.0406	0.2230	−0.18	0.856
Time (months)	0.00425	0.00969	0.44	0.667
Program (NTNP) × LIVE	−0.8690	0.3908	−2.22	0.027
Time × program (NTNP)	0.00905	0.01251	0.72	0.470
Time × LIVE	0.01958	0.01372	1.43	0.154
Time × program (NTNP) × LIVE	0.01788	0.02145	0.83	0.405

*Est* estimate, *SE* standard error

Using the site-level random intercept and slope terms, we conducted additional analysis to explore NTNP implementation effect at individual sites including plotting the observed monthly CCN and the predicted CCN from the model for each of the 11 NTNP sites and overlaying the mean observed month CCN for control sites (Supplementary Fig. 2). Individual contrasts were also conducted to compare each NTNP site to the corresponding time period in the control sites (Supplementary Table 5). These results indicate that 9 of the 11 sites significantly differed from control at the 0.05 level of significance but after adjusting for multiple comparisons this statistically significant difference remained for four NTNP sites.

## DISCUSSION

Our study demonstrates that within VHA, implementing a national-level Teleneurology outpatient clinical program in under-resourced, rural areas results in more timely neurologic care for Veterans and a significant decrease in the growth of non-VA CCN consultations after program implementation. Although neurology consultation rates increased slowly over time, both in VA and in community care, the drop in monthly CCN consults in NTNP sites compared to control sites was maintained throughout the period of this evaluation including up to 10 months post-implementation. We also found that wait times for scheduling and completing neurology visits were substantially lower for NTNP compared to community care.

Access to neurologists is a growing problem. Data from the National Provider Identifier^9^ system showed that only 3% of neurologists were located in rural counties, and only 20% of rural counties had at least one neurologist. Further, a recent study found that although the distribution of neurologists varies widely across various regions of the country, the prevalence of patients with neurologic conditions does not^10^, suggesting that significant unmet neurologic needs are more prevalent in rural regions of the USA. This disparity is one reason the American Academy of Neurology released a position paper focusing on the provision of Teleneurology care.

Although one could view access to care as binary (present/absent), it is important to consider other patient-centered aspects of the experience, namely patient and referring provider satisfaction. While there may be intuitive benefits to reduced wait times and travel times, the patient’s perspective, beliefs, and personal experience with virtual care may influence the longer-term sustainability and effectiveness of telehealth programs. In the NTNP program, we found that Veterans were highly satisfied with the Teleneurology service, and most would recommend Teleneurology care to other Veterans. We also found referring providers to be highly satisfied with NTNP consultations. This finding may not be surprising, since VHA has been an early adopter of many telehealth technologies, and nearly two-thirds of Veterans in our study reported having prior experience with telehealth as part of their routine care (data not shown). However, given that patients with neurologic conditions are older and may have specific cognitive or sensorimotor difficulties that impair use of telehealth technologies, we were reassured to find such high ratings of satisfaction and ease of use among our patients. We were similarly reassured that the referring providers also reported high levels of satisfaction with the teleneurology consultations and ongoing NTNP care.

In secondary analyses, we investigated which facilities had the most significant impact from NTNP implementation. We found that four of the 11 NTNP sites had significantly different community care consultation rates during their post-implementation period compared to all other control sites during that same period. Three of these four sites were among those with the smallest number of CCN consults, meaning that NTNP was likely able to address a greater proportion of their neurology consultation needs; however, other specific factors that might allow policymakers to target telehealth systems to sites most likely to benefit remain unknown. Additional qualitative analyses will explore site contextual factors related to successful NTNP implementation.

Limitations to our study include the relatively small number of facilities included and the variable time of post-implementation observation. As the number of sites was not a priori determined based on power to detect meaningful change, results should be interpreted with caution. Although the SARS-CoV-2 pandemic likely influenced referral patterns system-wide, the initial 7 months of the pandemic (March–October 2020) were part of the control period for all sites in this analysis and thus reduces the likelihood of variable pandemic influence on the results. Additionally, whether our findings would persist longer after implementation is not known; however, the maintenance of significantly lower CCN consult volumes over at least 10 months is promising. Additionally, we could only account for VA neurology FTE in a 1-year estimate, and we have no measure of local CCN availability, so we could not assess the impact of more granular changes in local VA or CCN availability during the study period which might impact referral patterns. For Veterans eligible for community care consultations, the use of NTNP was a choice; this may limit the generalizability of our satisfaction data to all Veterans (including those who chose CCN). We are conducting an ongoing project assessing Veteran satisfaction for stroke care across both NTNP and CCN which may provide additional information about Veteran preferences for Teleneurology care.

As the mismatch between the proportion of the population with neurologic conditions and the number of neurologists grows, Teleneurology outpatient care, as demonstrated by NTNP, is one means of improving patient access to neurologic specialists. This care overcomes some important barriers to access, including rural residence and scarcity of local neurologists, and can be provided with high satisfaction among patients and their referring primary care providers. Although little randomized trial evidence exists to assess the efficacy of telehealth care for chronic conditions, and most of this literature is in patients with diabetes^11^, our study suggests that Teleneurology care not only improves access to care but is feasible and acceptable to patients; future studies should assess the efficacy of Teleneurology care compared to in-person care for ongoing management of patients with neurologic conditions.

## Supplementary Information:

Below is the link to the electronic supplementary material.

Supplementary file1 (PDF 1055 KB)

Supplementary file2 (DOCX 274 KB)
