# Supplementary material for: The VA National Teleneurology Program (NTNP): Implementing Teleneurology to Improve Equitable Access to Outpatient Neurology Care
Source: J Gen Intern Med. 2023 Jun 20;38(Suppl 3):887–93. doi: 10.1007/s11606-023-08121-7 (PMC10356709; doi:10.1007/s11606-023-08121-7)
Supplement: Supplementary file 1 — Supplementary file1 (PDF 1055 KB) [file 11606_2023_8121_MOESM1_ESM.pdf]

Department of Veterans Affairs

Date: November 26, 2019

From: National Director of Neurology, Specialty Care Services (10P11)

Subject: Memorandum of Understanding (MOU) – Specialty Care Services and PRIS-M QUERI for Teleneurology Evaluation Amendment 1

To: PRIS-M QUERI Corresponding Principal Investigator, Roudebush VA Medical Center HSR&D Service (583)

1. PURPOSE: This MOU describes a formal partnership agreement between Specialty Care Services (Neurology) and the VA HSR&D Precision Monitoring Quality Enhancement Research Initiative (PRIS-M QUERI). Specialty Care Services is the Program Office responsible for implementing the VHA National Teleneurology Program. The Teleneurology Program's goal is to improve rural Veterans' access to neurologic care by developing a national telehealth program to provide coverage to under-resourced VA facilities which will lead to better care and outcomes for Veterans. Specialty Care Services has had ongoing, productive partnerships with Indianapolis PRIS-M QUERI investigators through the work on evaluating the VA National Telestroke Program, scheduled to be completed in FY2020.

As specified in the Project Agreement (Attachment 1), work will be conducted in collaboration with Specialty Care Services to plan and conduct an ongoing evaluation of the Teleneurology Program using resources and specific expertise of the PRIS-M QUERI. An outline of the proposed evaluation and annual budget is provided in Attachment 2.

2. GENERAL STRUCTURE OF PRODUCT: Attachment 1 provides a preliminary outline of the initial scope of work that will launch the program evaluation and collaboration between Specialty Care Services and the PRIS-M QUERI. Attachment 2 summarizes the initial aims of the collaboration, the proposed methods, data sources, and outcomes of the evaluation; and key collaborators and estimated budget request over the 3-year period. Senior leaders of both Specialty Care Services and the PRIS-M QUERI will review the planned scope of work for suitability and importance. Documentation of review and discussion will be captured in meeting minutes which will be kept on file by Specialty Care Services and the PRIS-M QUERI. Annual review and approval of proposed scope of work for the next year will be conducted by the Office of Specialty Care/Office of Rural Health.

3. RESPONSIBILITIES: In this partnership, Specialty Care Services and PRIS-M will collaborate to carry out the Teleneurology Program evaluation. Either Specialty Care Services or PRIS-M may propose specific partnership methods or aims, but Specialty Care Services will retain exclusive right of determining whether the specific proposal supports internal VA purposes (i.e., is both aligned with VHA priorities and supports VHA health operational needs).

4. SCOPE/COST:

a) The cost of the MOU will cover the three-year evaluation beginning in FY20 and ending in FY23. Funding will be approximately $180,000 in any year, unless additional amendments are approved by the Office of Specialty Care.

b) This MOU must be amended each year with an update to the proposed budget for that year, including confirmation and/or change of any personnel.

c) The proposed three-year Project Budget is provided in Attachment 2.

5. TERMS OF MOU: The MOU period will be effective 01/01/2020-09/30/2023.

6. EMPLOYEE RESPONSIBILITIES: Employees are responsible for adhering to all applicable Roudebush VA Medical Center policies and practices to include timekeeping, travel, security, etc.

7. Specialty Care Services (10P11) RESPONSIBILITIES: Cost transfer – Specialty Care Services will direct the Office of Rural Health (funding office) to execute annual expenditure transfers to Roudebush VA Medical Center (583) to cover current gross pay with benefits for employees listed in this MOU based on FTE specified.

8. Roudebush VA Medical Center (583) RESPONSIBILITIES:

a) Primary duty station for employees listed in this MOU remains Roudebush VA Medical Center.
b) Duty and leave status, including timecard processing, for the employee, personnel actions, performance appraisals will be handled by Roudebush VA Medical Center (583).
c) Staff designated will receive all logistical support (e.g., personnel, ETA, payroll, security [badges, key cards], performance plans and appraisals, IT equipment and support, access to local VISN and facility networks, etc.) from Roudebush VA Medical Center (583).

9. TERMS OF AGREEMENT: AMENDMENTS AND TERMINATION: This MOU may be terminated by either Specialty Care Services or PRIS-M. The terms of this MOU may be amended by mutual agreement of both parties upon thirty (30) calendar days of notification. All requests for amendments must be made in writing and signed by both parties. Reasons for amendment may include additions or changes in designated personnel. This MOU may be terminated at the close of a fiscal year by either party with ninety (90) calendar days of written notification. Such cancellation will be executed only by the signature approval of the parties to this agreement or their designees or official successors.

In the event the MOU is terminated, PRIS-M will be paid through the deliverable completed.

10. CONTACTS: The Specialty Care Services administrative contact for this MOU is the TeleNeurology Program administrator. The Roudebush VA Medical Center administrative contact for this MOU is Jennifer Myers, 317-988-4407.

________________________________ 12/02/2019

Donald Higgins, MD — Date
National Program Director for Neurology
Specialty Care Services (10P11), VA Central Office, Washington, DC

________________________________ 11/26/2019

Linda Williams, MD — Date
PRIS-M QUERI Co-Principal Investigator, Roudebush VAMC HSR&D Service

________________________________ 11/26/2019

Teresa Damush, PhD — Date
PRIS-M QUERI Corresponding Principal Investigator, Roudebush VAMC HSR&D Service

________________________________ 12-3-19

Laura E. Ruzick, FACHE — Date
Acting Medical Center Director, Richard L. Roudebush VAMC

Attachment 1
TeleNeurology Evaluation Scope of Work

Initial work proposed:

1. Characterize the unmet neurology needs at TeleNeurology participating facilities and/or candidate facilities including:
   a. Neurology access in the year prior to TeleNeurology program implementation
   b. Local neurology wait times, and
   c. Volume/costs of community care Neurology consultation
2. Evaluate the implementation and impact of the VA TeleNeurology program.

Evaluation Specific Aims:
Using the RE-AIM framework, we propose the following specific aims for the Teleneurology evaluation.

1. Assess program <u>reach</u> among all eligible VA facilities, and at participating facilities among Veterans for whom a neurology consultation is requested.
2. Assess program <u>effectiveness</u> by measuring key system, provider, and patient outcomes; compare the effectiveness between participating sites
3. Assess program <u>adoption</u> by participating facilities by identifying barriers and facilitators to program implementation and sustainment; and among participating Teleneurology providers by monitoring satisfaction among Teleneurology providers
4. Monitor and characterize <u>implementation</u> consistency, adaptations, and costs by developing systems to efficiently track implementation progress at participating sites, assess barriers and facilitators to implementation, and by assessing ongoing facility-level costs of implementation and of non-VA outpatient neurology care.
5. Examine program <u>maintenance</u> over time at participating facilities by monitoring and recording efforts to secure buy-in at participating facilities and by conducting interviews with facility leadership and national program staff to describe plans for program continuation and support.

Evaluation Data Sources and Methods:
The TeleNeurology Evaluation is an operational/quality improvement project; official designation of the project as such will be obtained prior to starting the evaluation. TeleNeurology Evaluation staff will obtain required permissions for access to necessary data sources and other VA data systems prior to conducting any project work. Data requirements, sources, and systems may change based on program needs; proposed data sources likely to be used in this evaluation include:

1. VSSC: Encounter, workload, access, and cost data
2. CDW: Patient-specific data, TIU notes, health factors
3. HERC Labor Cost Data and Average Cost Data: estimated staff and outpatient encounter costs
4. Cerner data: If participating facilities migrate to Cerner during the time period of the Evaluation, we will work with TeleNeurology Program staff to assess data sources and availability of key evaluation data obtained from CDW and other VA sources.

TeleNeurology Evaluation staff will conduct Veteran and VA staff interviews at participating sites, as well as interviews with TeleNeurology program partners according to the following proposed schedule. Interview content and final schedule will be determined in consultation with the TeleNeurology Program and Evaluation Leadership.

Interviewee	Method	Timeframe
Teleneurology Program leadership and staff	Telephone or in-person	Annually
National Teleneurology Partners	Telephone	Annually
Participating facility staff (operational and clinical leaders, staff engaged with Teleneurology implementation)	In-person	First six months of program implementation<br>Last year of program participation
Veterans offered or completing a Teleneurology consultation	Telephone	Within two weeks of completed consultation, within two weeks of a missed consultation

Evaluation Outcomes
Evaluation Outcomes are defined in the accompanying TeleNeurology Evaluation Plan document (Appendix 2). Outcome definitions are subject to change based on program needs and existing data sources. Evaluation outcomes will follow the RE-AIM framework regardless of modifications in the program design or data sources. Key questions addressed by the planned evaluation outcomes include:

1. Reach
    a. Did the program reach intended target population (facility- and patient-level)?
    b. How many within the intended population or what proportion of this population participated?
    c. What approaches were used to reach the target population?
2. Effectiveness
    a. What is the effectiveness of TeleNeurology at the system-, provider- and patient-level?
    b. Does Teleneurology effectiveness vary between locations of implementation?To what degree did the participating facilities or the target population engage in the program?
3. Adoption
    a. Were there unanticipated obstacles that prevented sites from adequate engagement? What methods were employed to overcome obstacles?
    b. Did the selected sites for enterprise-wide implementation operation or expansion prove to be appropriate?
    c. To what degree did the participating facilities or the target population engage in the program?
4. Implementation
    a. Did sites differ in implementation, and if so, why?
    b. Was the intervention delivered with fidelity to the program's core elements and goals?
    c. Was effectiveness impacted by differences in fidelity, and to what degree?
    d. What barriers were encountered and how were they addressed?
    e. What facilitators were identified and how did facilities increase implementation success?
    f. What specific strategies are required to ensure successful program implementation?

5. Maintenance
    a. What proportion of sites sustain program participation? Are TeleNeurology FTE transferred to the VISN Telehealth Specialty Care Clinical Resource Hubs?
    b. What plans were developed to incorporate Teleneurology into ongoing operational practices, so that it will be delivered over the longer term?
    c. What efforts were required to secure buy-in by facility leadership and key staff at each site. What were site leaders most concerned about with the Teleneurology program and what were they most excited about?
    d. What efforts took place to secure buy-in by national leadership and key staff?
    e. What planning has been done towards continuation of successful programs once ORH funding ends?

Additional Evaluation outcomes will be analyzed in conjunction with input from TeleNeurology program leadership including:

- Improvements in access to Neurology over time (pre- versus post-TeleNeurology implementation) at participating facilities
- Business case analysis for the TeleNeurology program
- Other standard program data and milestones as required

Evaluation Deliverables:

1. Quarterly reports beginning the first full quarter after initial TeleNeurology active implementation:
    a. Key RE-AIM evaluation outcomes (overall and by participating facility)
    b. Descriptive data for participating facilities (type of consultation, diagnoses, Veteran characteristics)
    c. Other data as agreed upon by TeleNeurology and Evaluation leadership
2. Annual report of qualitative data
    a. Veteran interviews
    b. VA staff interviews
    c. Implementation progress report
    d. Other annual data as agreed upon by TeleNeurology and Evaluation leadership

Key Evaluation Team Members: Linda S. Williams, MD (Evaluation Director); Holly Martin, MPH (Evaluation Project Manager); Teresa M. Damush, PhD (Implementation Science Lead); Laura J. Myers, PhD (Data Scientist); Jessica Coffing, MPH (Data Manager)

Estimated resources:
See attached budget for FY2020-FY2022

VA Teleneurology Evaluation Proposal: Draft

Overall Objective: The VA Teleneurology Program proposes to improve rural Veterans' access to outpatient neurologic care by developing a national neurology telehealth program to provide coverage to under-resourced VA facilities using a bi-coastal hub-spoke model. This program directly addresses the known national shortage of general neurology physician staff in the VHA healthcare system.

The proposed VA Teleneurology Program Evaluation draws on the experience of the Precision-Monitoring (PRIS-M) QUERI in evaluation and implementation of telehealth-based Specialty Care programs, especially those focusing on neurologic and rehabilitation care in VHA. Under the leadership of Dr. Linda Williams, neurologist and PRIS-M QUERI Co-PI, the PRIS-M QUERI is leading the evaluation of the VA National Telestroke Program, scheduled to be completed in FY 2020. This experience will be helpful to the Teleneurology Program as we work with VA Teleneurology Program, Neurology Specialty Care, and Office of Rural Health representatives to develop and implement a Teleneurology Evaluation to measure the effectiveness of program implementation and the impact of implementation on VA care, staff both requesting and providing neurology consultation, and on Veterans' care and outcomes.

A. Initial Work Proposed

The PRIS-M QUERI will work with Specialty Care-Neurology partners to:

1. Characterize the unmet neurology needs at participating facilities including: Neurology access measures in the year prior to program implementation, local neurology consultation wait times and volume and cost of community care Neurology consultation.
2. Evaluate the implementation and impact of the VA Teleneurology program.

B. Evaluation Specific Aims

Using the RE-AIM framework, we propose the following specific aims for the Teleneurology evaluation.

1. Assess program reach among all eligible VA facilities, and at participating facilities among Veterans for whom a neurology consultation is requested.
2. Assess program effectiveness by measuring key system, provider, and patient outcomes; compare the effectiveness between participating sites
3. Assess program adoption by participating facilities by identifying barriers and facilitators to program implementation and sustainment; and among participating Teleneurology providers by monitoring satisfaction among Teleneurology providers
4. Monitor and characterize implementation consistency, adaptations, and costs by developing systems to efficiently track implementation progress at participating sites, assess barriers and facilitators to implementation, and by assessing ongoing facility-level costs of implementation and of non-VA outpatient neurology care.
5. Examine program maintenance over time at participating facilities by monitoring and recording efforts to secure buy-in at participating facilities and by conducting interviews with facility leadership and national program staff to describe plans for program continuation and support.

C. Data Sources and Methods

PRIS-M QUERI investigators will work with Teleneurology and Specialty Care-Neurology leadership to plan data acquisition and analysis responsibilities for this evaluation. Data requirements and analyses may change based on program needs and/or changes in underlying program data, data structures, or effort.

Proposed data sources likely to be used in this evaluation include:

1. VSSC: Encounter, workload, access, cost data. Some of these data may be accessed by Teleneurology program staff and some by Evaluation staff.
2. CDW: Patient demographics, comorbidity, diagnoses; consultation and visit data. If templated notes with health factors are developed, these health factors relevant to the evaluation will also be obtained.
3. HERC Labor Cost Data, Allocation Resource Center and Average Cost Data: estimate the cost of staff time for hub and at spoke sites; estimate outpatient encounter costs; estimate VERA cost returns
4. Patient Interviews: Patient technology ratings; telepresence ratings; satisfaction; recommendations.
5. Participating site provider and administrator interviews and surveys: Organizational Readiness to Change Assessment; satisfaction; adoption; implementation barriers, facilitators, and outcome; maintenance outcomes.
6. Participating teleneurologist interviews and surveys: satisfaction; implementation barriers, facilitators, and outcomes.
7. Implementation activity monitoring and tracking
    a. PRIUS tool: track ongoing implementation barriers, facilitators, successes at hub and spoke levels
    b. Interviews with spoke administrators and providers pre- and post-implementation

We will prepare quarterly reports on an agreed-upon set of standard program metrics, and an annual report with additional program data.

We will work with Teleneurology Program leadership to develop standardized interviews to capture key program and implementation data and outcomes as described in sections below according to the following schedule:

Interviewee	Method	Timeframe
Teleneurology Program leadership and staff	Telephone or in-person	Annually
National Teleneurology Partners	Telephone	Annually
Participating facility staff (operational and clinical leaders, staff engaged with Teleneurology implementation)	In-person	First six months of program implementation at each site Last year of program participation
Veterans offered or completing a Teleneurology consultation	Telephone	Within two weeks of completed consultation, within two weeks of a

		missed or declined consultation

D. Proposed Program Outcomes

1. **<u>Reach Outcomes</u>**

The Teleneurology Program and Evaluation teams will work together to define the target population (facilities potentially eligible to implement Teleneurology) using existing definitions and data sources (e.g. VHA Rurality calculator, SPARQ/access data, OCC cost data) including:

- >50% rurality VAMCs
- Outpatient neurology access >28 days
- VAMCs with high Community Care (CC) neurology costs
- Veterans in areas with neither VAMC or CC neurological care

Reach outcomes will be captured at the facility and the patient level (including reporting counts of services to rural and non-rural Veterans) using specific denominator and numerators as defined below to address the following key questions:

1a. Did the program reach intended target population?

- Facility-level reach:
  - Denominator:
    - D0: Number of facilities meeting eligibility criteria for joining the Teleneurology program
  - Numerator:
    - N0: Number of facilities that successfully implement Teleneurology

- Patient-level reach:

  - Denominators:
    - D1: Veterans referred for or offered Teleneurology consultation
    - D2: Veterans referred for or offered community care neurology consultation
    - D3: Veterans referred for or offered local VA neurology consultation (if available)
    - D4: Sum of D1 and D2 and D3
  - Numerators:
    - N1: All Veterans scheduled for a Teleneurology consultation
    - N2: All Veterans completing a Teleneurology consultation
    - N3: Number of Teleneurology new patient encounters
    - N4: Number of Teleneurology follow-up encounters

We will similarly assess the number of Veterans referred for, scheduled for, and completing. a community care neurology consultation.

1b. How many within the intended population or what proportion of this population participated?

- Denominators:
  - D5: Number of projected new patient encounters based on Teleneurology FTE
  - D6: Number of projected follow-up patient encounters based on Teleneurology FTE
  - D7: Total projected Teleneurology encounters: Sum of D5 and D6
- Numerators:
  - N3: Number of Teleneurology new patient encounters
  - N4: Number of Teleneurology follow-up encounters

1c. What approaches were used to reach the target population?

- Approaches by the Teleneurology program to identify and recruit spoke sites will be identified by the Evaluation Team using the PRIUS tool as applied to ongoing program teleconferences, meetings, and individual interviews. Any methods used by participating facilities to identify or select Veterans eligible for Teleneurology will be identified during site interviews.

2. **Effectiveness Outcomes**

The evaluation will include effectiveness outcomes at the system, provider, and patient levels to address the following questions:

2a. What is the effectiveness of TeleNeurology at the system-, provider- and patient-level?

- System-level outcomes (assessed for Teleneurology and community care neurology consultations using existing VA data systems):
  - Time from consultation placement to scheduled consultation
  - Time from consultation placement to completed consultation
  - Consultation completion, cancellation and no-show rates

- Provider-level outcomes (interviews):
  - Spoke provider satisfaction with Teleneurology
  - Spoke administrator satisfaction with Teleneurology
  - Teleneurology provider satisfaction

- Patient-level outcomes (interviews):
  - Patient satisfaction
    - We will include Veteran caregiver satisfaction if a caregiver was present for the consultation and is present at the time of the telephone interview

- We will interview Veterans that schedule but do not complete a Teleneurology consultation to identify barriers to Teleneurology and explore the range of Veteran attitudes toward Teleneurology care

2b. Does Teleneurology effectiveness vary between locations of implementation?

We will compare system, provider, and patient outcomes within sites over time and between sites that implement Teleneurology. We will also analyze qualitative data from interviews to categorize variation in implementation and identify factors related to successful implementation of Teleneurology

3. **<u>Adoption Outcomes</u>**

We will conduct scheduled, structured interviews with Teleneurology leadership, participating facility staff, and representatives of national partner offices to address questions related to Teleneurology Program adoption as described below.

3a. Were there unanticipated obstacles that prevented sites from adequate engagement? What methods were employed to overcome obstacles?

- We will conduct annual interviews with the Teleneurology leadership team and administrators to identify key barriers and facilitators to developing program organizational support and to program adoption.
- We will interview participating facility leadership and staff in the first six months of program implementation and in the final year of the program (or at the time the facility exits the program), including facility operational and clinical staff, Telehealth Coordinators and providers interacting with Teleneurology to understand site barriers and facilitators to adoption of the program.
- We will interview representatives from national (Office of Rural Health, Office of Specialty Care Services, Office of Connected Care) and regional (VISN Clinical Resource Hubs) offices that are active operational partners for ongoing Teleneurology activities in the initial and the final year of the program to identify obstacles and solutions to program development and adoption at the national level.
- We will also track ongoing implementation activities related to adoption of the Teleneurology program at the national level and among sites by participating on Teleneurology program calls and using the PRIUS tool developed in other PRIS-M QUERI projects to systematically track and categorize direction and magnitude of the impact of these activities.

3b. Did the selected sites for enterprise-wide implementation operation or expansion prove to be appropriate?

We will compare facility information on Neurology wait times and number of local Neurology FTE among those that participate in Teleneurology and those that do not.

3c. To what degree did the participating facilities or the target population engage in the program?

Not all facilities that qualify to implement Teleneurology will select to implement Teleneurology. We will monitor the engagement of the target population at the system and the patient level as follows:

- System-level:
  - Denominator: D8: Number of sites offered participation in Teleneurology
  - Numerator: N0: Number of facilities that successfully implement Teleneurology

We will also track reasons that eligible sites do not participate as discussed during Teleneurology program meetings/calls.

- Patient-level (overall and by participating facility):

  We will track the proportion of all neurology consultations (local neurology, Teleneurology and community care neurology consultations) at a given facility that are completed via Teleneurology. We recognize that the “optimal” proportion will differ between facilities based on local VA and non-VA resources, current access metrics, and by volume so we do not anticipate comparing facilities. By tracking at the patient and the facility level, however, we can better understand how Teleneurology resources are being used and identify if there are sites where engagement barriers may need to be further explored.

4. **Implementation Outcomes**

We will work with the Teleneurology Program team to develop a standardized checklist of activities that each facility will need to implement Teleneurology and will assist program staff in using the SiPREP tool, developed and used in other PRIS-M QUERI projects, to track each facility’s progress on implementation steps.

We will track implementation fidelity to the planned strategy and changes in implementation strategies over time by participating in Teleneurology program meetings and calls. The Evaluation Team will use the PRIUS tool to record observations during these meetings to systematically record the direction and magnitude of factors influencing program implementation.

We will also conduct interviews with Teleneurology program leadership and facility staff interviews during the first six months of program implementation and during the last year of the program, or at the time a facility exits the program as described above, to obtain additional qualitative input on implementation fidelity, strategies, and changes.

These interviews will allow us to address the following questions:

- Did sites differ in implementation, and if so, why?
- Was the intervention delivered with fidelity to the program's core elements and goals?
- Was effectiveness impacted by differences in fidelity, and to what degree?
- What barriers were encountered and how were they addressed?
- What facilitators were identified and how did facilities increase implementation success?
- What specific strategies are required to ensure successful program implementation?

5. **<u>Maintenance Outcomes</u>**

We will monitor the proportion of sites that sustain participation in the program (through the end of the evaluation period or until their need for temporary neurology consultation is met) as defined:

- Denominator:
  - N0: Number of facilities that successfully implement Teleneurology
- Numerator:
  - N5: Number of facilities that sustain participation in Teleneurology

We will also track the number of TN FTE that are transferred to the VISN Telehealth Specialty Care Clinical Resource Hubs as a measure of TN sustainability and spread.

During annual interviews with the Teleneurology program leadership/staff we will address the following questions to synthesize experiences and provide iterative input into the evaluation:

- What plans were developed to incorporate Teleneurology into ongoing operational practices, so that it will be delivered over the longer term?
- What efforts were required to secure buy-in by facility leadership and key staff at each site. What were site leaders most concerned about with the Teleneurology program and what were they most excited about?
- What efforts took place to secure buy-in by national leadership and key staff?
- What planning has been done towards continuation of successful programs once ORH funding ends?

6. **<u>Other Program Measures</u>**

In addition to the formal evaluation measures outlined above, additional data will be captured by the Teleneurology Program team and/or the Evaluation team as described below.

<u>6a. Improvement in access</u>

We will use existing VA access data in the year prior to Teleneurology implementation at each site to compare changes in Veteran wait times to access neurological care and reduction in “no-shows” (missed opportunities). The Teleneurology team will conduct chart review on a sample of cases to ensure data associated with consult completion at each site are accurate.

6b. Business case analysis

The Evaluation Team will work with Teleneurology leadership in the first year of the program to plan a business case analysis. We will consult with HERC leadership on this plan to ensure we are capturing the necessary data at the program and facility levels. Candidate cost data and estimates will include:

- Estimated cost of program hub FTE
- Estimated cost (hours) of spoke site staff FTE for implementation
- Estimated cost (hours) of spoke site staff FTE for ongoing consultation support
- Spoke site community care neurology annual costs
- Estimated Veteran travel cost savings (based on Veteran eligibility for travel pay and zip code of residence)

6c. Standard program milestones

The Teleneurology program team will also track additional standard milestones for the program including:

- Overall number of funded FTE working on project
- Overall number of vacant FTE
- Contracts
- Equipment costs
- Project Operational costs
- Additional Workforce Training and Educational Standard Measures (number of clinicians trained, number of non-clinical staff trained)
