# Supplementary material for: The VA National Teleneurology Program (NTNP): Implementing Teleneurology to Improve Equitable Access to Outpatient Neurology Care
Source: J Gen Intern Med. 2023 Jun 20;38(Suppl 3):887–93. doi: 10.1007/s11606-023-08121-7 (PMC10356709; doi:10.1007/s11606-023-08121-7)
Supplement: Supplementary file 2 — Supplementary file2 (DOCX 274 KB) [file 11606_2023_8121_MOESM2_ESM.docx]

**NTNP Access Supplementary Analyses**

Supplementary Figure 1. Pre-implementation and post-implementation time periods for NTNP and control sites

**First NTNP go-live date**

|  | site | 1 | 2 | 3 | 4 | 5 | 6 | 7 | 8 | 9 | 10 | 11 | 12 | 13 | 14 | 15 | 16 | 17 | 18 | 19 | 20 | 21 | 22 | 23 | 24 |
| --- | --- | --- | --- | --- | --- | --- | --- | --- | --- | --- | --- | --- | --- | --- | --- | --- | --- | --- | --- | --- | --- | --- | --- | --- | --- |
| NTNP | 1 |  |  |  |  |  |  |  |  |  |  |  |  |  |  |  |  |  |  |  |  |  |  |  |  |
|  | 2 |  |  |  |  |  |  |  |  |  |  |  |  |  |  |  |  |  |  |  |  |  |  |  |  |
|  | 3 |  |  |  |  |  |  |  |  |  |  |  |  |  |  |  |  |  |  |  |  |  |  |  |  |
|  | 4 |  |  |  |  |  |  |  |  |  |  |  |  |  |  |  |  |  |  |  |  |  |  |  |  |
|  | 5 |  |  |  |  |  |  |  |  |  |  |  |  |  |  |  |  |  |  |  |  |  |  |  |  |
|  | 6 |  |  |  |  |  |  |  |  |  |  |  |  |  |  |  |  |  |  |  |  |  |  |  |  |
|  | 7 |  |  |  |  |  |  |  |  |  |  |  |  |  |  |  |  |  |  |  |  |  |  |  |  |
|  | 8 |  |  |  |  |  |  |  |  |  |  |  |  |  |  |  |  |  |  |  |  |  |  |  |  |
|  | 9 |  |  |  |  |  |  |  |  |  |  |  |  |  |  |  |  |  |  |  |  |  |  |  |  |
|  | 10 |  |  |  |  |  |  |  |  |  |  |  |  |  |  |  |  |  |  |  |  |  |  |  |  |
|  | 11 |  |  |  |  |  |  |  |  |  |  |  |  |  |  |  |  |  |  |  |  |  |  |  |  |
| Control | 1 |  |  |  |  |  |  |  |  |  |  |  |  |  |  |  |  |  |  |  |  |  |  |  |  |
|  | 2 |  |  |  |  |  |  |  |  |  |  |  |  |  |  |  |  |  |  |  |  |  |  |  |  |
|  | 3 |  |  |  |  |  |  |  |  |  |  |  |  |  |  |  |  |  |  |  |  |  |  |  |  |
|  |  |  |  |  |  |  |  |  |  |  |  |  |  |  |  |  |  |  |  |  |  |  |  |  |  |
|  | 7 |  |  |  |  |  |  |  |  |  |  |  |  |  |  |  |  |  |  |  |  |  |  |  |  |

Blue represents the baseline period prior to NTNP implementation, Red represents the months of data used in the analysis for the post-NTNP period. Two NTNP sites went live in month 13 but in the last half of the month, so month 13 was included in the pre-implementation period for all sites. Control sites used all the second fiscal year as the post program time-period, NTNP sites used all the months after each individual site went live with the NTNP. White represents months not included in the analysis.

Supplementary Table 1. Site-level descriptive statistics before and after first site went live with NTNP. N is the number of months of data in the period, and the mean/median values indicate monthly CCN neurology consults in the period.

|  | Pre-NTNP | | | Post-NTNP | | |  |
| --- | --- | --- | --- | --- | --- | --- | --- |
| NTNP sites | N | Mean (SD) | Median (IQR) | N | Mean (SD) | Median (IQR) |  |
| Site 1 | 13 | 20.8 (8.6) | 20 (13) | 11 | 31.5 (7.8) | 29 (12) |  |
| Site 2 | 13 | 21.6 (5.3) | 21 (4) | 11 | 17.8 (5.0) | 19 (08) |  |
| Site 3 | 13 | 20.4 (6.0) | 19 (6) | 8 | 14.0 (6.1) | 15 (10) |  |
| Site 4 | 13 | 52.7 (14.1) | 55 (16) | 8 | 58.9 (13.0) | 60 (15) |  |
| Site 5 | 13 | 29.8 (9.4) | 27 (13) | 8 | 27.9 (8.3) | 29 (13) |  |
| Site 6 | 13 | 45.9 (14.4) | 54 (19) | 7 | 56.7 (15.3) | 61 (24) |  |
| Site 7 | 13 | 94.9 (20.1) | 91 (25) | 6 | 87.2 (19.3) | 84 (30) |  |
| Site 8 | 13 | 30.9 (7.3) | 29 (11) | 5 | 36.0 (8.9) | 36 (07) |  |
| Site 9 | 13 | 47.2 (12.9) | 50 (12) | 5 | 32.6 (6.7) | 36 (10) |  |
| Site 10 | 13 | 38.0 (16.2) | 36 (20) | 4 | 64.8 (6.1) | 65 (11) |  |
| Site 11 | 13 | 109.4 (23.5) | 114 (15) | 4 | 135.5 (10.8) | 134 (14) |  |
|  | Pre-NTNP | | | Post-NTNP | | |  |
| Control sites | N | Mean (SD) | Median (IQR) | N | Mean (SD) | Median (IQR) |  |
| Site 12 | 13 | 46.0 (14.9) | 42 (27) | 11 | 107.7 (55.9) | 102 (116) |  |
| Site 13 | 13 | 26.2 (7.6) | 25 (10) | 11 | 45.6 (12.2) | 50 (20) |  |
| Site 14 | 13 | 39.6 (11.7) | 38 (10) | 11 | 64.8 (15.1) | 63 (28) |  |
| Site 15 | 13 | 102.2 (27.9) | 109 (34) | 11 | 144.0 (19.6) | 154 (34) |  |
| Site 16 | 13 | 62.8 (19.1) | 57 (16) | 11 | 66.3 (21.4) | 63 (22) |  |
| Site 17 | 13 | 35.6 (12.8) | 32 (15) | 11 | 45.3 (14.6) | 37 (28) |  |
| Site 18 | 13 | 91.5 (39.9) | 67 (55) | 11 | 101.1 (35.1) | 102 (65) |  |

Supplementary Table 2. Model Fit Characteristics

| Model | Poisson | Negative Binomial |
| --- | --- | --- |
| # parameters | 11 | 12 |
| -2LL | 4051.65 | 3246.78 |
| AIC | 4073.65 | 3270.78 |
| Pearson Chi-Square/DF | 4.36 | 0.91 |

Supplementary Table 3. Covariance Parameter Estimates (NB Model), random site-level intercept and slope and scale parameter of NB.

| Parameter | Estimate | SE |
| --- | --- | --- |
| Intercept | .2479 | .0842 |
| Time | .00019 | .00009 |
| Scale | .0695† | .007 |

†The estimated conditional variance of the count is $\hat{\mu}_{i}+.0695\hat{\mu}_{i}^{2}$, SE = Standard Error.

Supplementary Table 4. Contrasts obtained from negative binomial model

| Effect | Est | SE | t Value | p-value| | Mean [95% CI] |
| --- | --- | --- | --- | --- | --- |
| Control sites pre-time period | 4.071 | 0.212 | 19.17 | <.0001 | 58.6 [38.6, 89.0] |
| Control sites post-time period | 4.453 | 0.230 | 19.34 | <.0001 | 85.9 [54.6, 135.1] |
| Control sites post-pre | 0.383 | 0.077 | 4.96 | <.0001 | 1.5 [1.3, 1.7] |
| NTNP sites pre-time period | 3.779 | 0.166 | 22.71 | <.0001 | 43.8 [31.5, 60.7] |
| NTNP sites post-time period | 3.741 | 0.190 | 19.68 | <.0001 | 42.1 [29.0, 61.2] |
| NTNP sites post-pre | -0.038 | 0.074 | -0.52 | 0.603 | 1.0 [0.8, 1.1] |
| (NTNP post-pre)-(control post-pre) | -0.421 | 0.107 | -3.93 | 0.0001 | 0.7 [0.5, 0.8] |

Est = Estimate, SE = Standard Error

Supplementary Figure 2. NTNP site Community Care Consults compared to Control sites.

For each individual NTNP Site 1-11, these graphs show the number of Community Care Neurology consults by month (black points), mean monthly Community Care Neurology consults for control sites (red points), and predicted CCN consults from model (black line), over the study period.


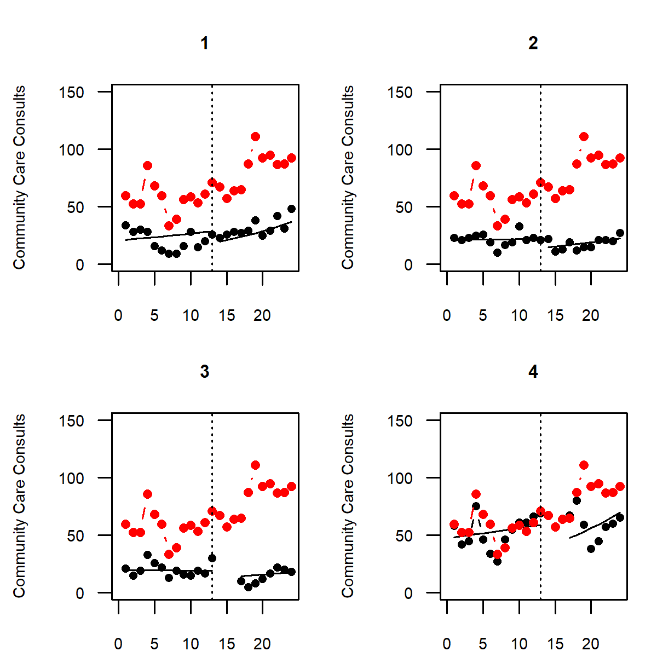

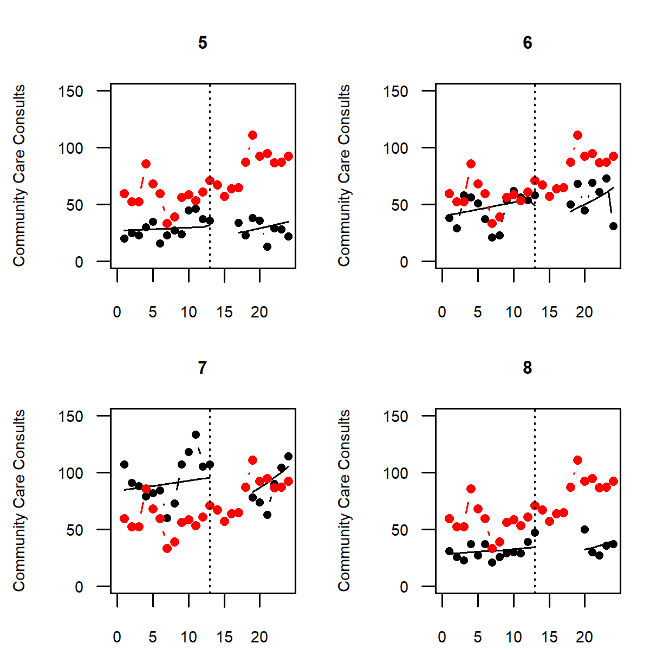


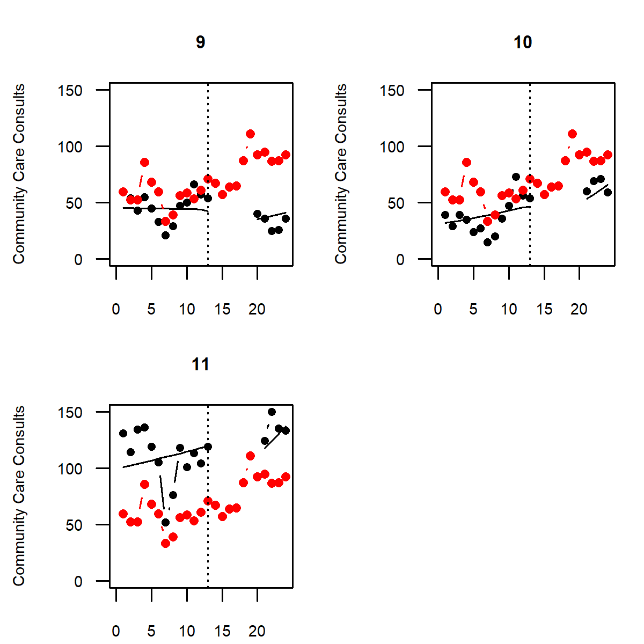


Supplementary Table 5. Site-level contrasts comparing NTNP sites to identical time span in control sites.

| Contrast | EST | SE | t Value | p-value | Significant Contrasts  Among NTNP sites |
| --- | --- | --- | --- | --- | --- |
| (**Site 1** post-pre)-(control sites post-pre) | -0.2793 | 0.1291 | -2.16 | 0.0312 |  |
| (**Site 2** post-pre)-(control sites post-pre) | -0.5443 | 0.1313 | -4.15 | <.0001 | * |
| (**Site 3** post-pre)-(control sites post-pre) | -0.6172 | 0.1495 | -4.13 | <.0001 | * |
| (**Site 4** post-pre)-(control sites post-pre) | -0.3298 | 0.1343 | -2.45 | 0.0146 |  |
| (**Site 5** post-pre)-(control sites post-pre) | -0.4360 | 0.1407 | -3.10 | 0.0021 | * |
| (**Site 6** post-pre)-(control sites post-pre) | -0.1843 | 0.1604 | -1.15 | 0.2514 |  |
| (**Site 7** post-pre)-(control sites post-pre) | -0.4020 | 0.1431 | -2.81 | 0.0053 |  |
| (**Site 8** post-pre)-(control sites post-pre) | -0.3163 | 0.1564 | -2.02 | 0.0439 |  |
| (**Site 9** post-pre)-(control sites post-pre) | -0.5757 | 0.1638 | -3.51 | 0.0005 | * |
| (**Site 10** post-pre)-(control sites post-pre) | -0.0245 | 0.1692 | -0.14 | 0.8852 |  |
| (**Site 11** post-pre)-(control sites post-pre) | -0.3156 | 0.1549 | -2.04 | 0.0423 |  |
